# International consensus on adolescent metabolic health: prevention of obesity and type 2 diabetes

**DOI:** 10.1186/s13098-026-02142-y

**Published:** 2026-04-18

**Authors:** Inass Shaltout, Peter Egbert Hermann Schwarz, Amr El-Meligi, Iman El Sherif, Hany Hammad, Shereen Abdelghaffar, Mona Hegazy, Gamela Nasr, Heba Habeeb, Engy Barakat, Tatjana Milenkovic, Andrew J. Behnke, Mohamed Hassanein, Jelica Bjekic Macut, Rajeev Chawla, Shalini Jaggi, Jamal Belkadir, Dario Rahelić, Diop Said Norou, Florian Toti, Amin Roshdy Soliman

**Affiliations:** 1 Internal Medicine and Diabetes Department, Faculty of Medicine, Cairo University, Cairo, Egypt; 2 Department for Prevention and Care of Diabetes, Faculty of Medicine, Carl Gustav Carus at the Technische Universität/TU Dresden, Dresden, Germany; 3 Faculty of Medicine, Paul Langerhans Institute Dresden of Helmholtz Zentrum München at University Hospital, TU Dresden, Germany; 4 Department of Medicine III, Prevention and Care of Diabetes, German Center for Diabetes Research, University of Dresden, Dresden, Germany; 5 Internal Medicine and Diabetes Department, Faculty of Medicine, Cairo University, Cairo, Egypt; 6 Internal Medicine Department, Endocrinology & Diabetes, Faculty of Medicine, Suez Canal University, Ismailia, Egypt; 7 Internal Medicine and Nephrology Department, Faculty of Medicine, Cairo University, Cairo, Egypt; 8 Department of Pediatrics, Pediatric Diabetes and Endocrinology Unit, Faculty of Medicine, Cairo University, Cairo, Egypt; 9 Internal Medicine Department, Faculty of Medicine, Cairo University, Cairo, Egypt; 10 Department of Cardiovascular Medicine, Suez Canal University, Cairo, Egypt; 11 Faculty of Medicine, University Clinic for Endocrinology, Diabetes and Metabolic Disorders, University Clinic for Endocrinology, Ss. Cyril and Methodius University in Skopje, Skopje, 1000 Republic of Macedonia; 12 Chief Section of Endocrinology, Virginia Tech Carilion School of Medicine, Roanoke, VA USA; 13 Department of Diabetes and Endocrinology, Mohamed Bin Rashid University, Dubai, UAE; 14 Department of Endocrinology, Faculty of Medicine, University Hospital Medical Center, University of Belgrade, Bezanijska kosa, Belgrade, Serbia; 15 North Delhi Diabetes Centre, DIPSI, New Delhi, India; 16 Lifecare Diabetes Centre, New Delhi, India; 17 Department of Endocrinology and Diabetes, Faculty of Medicine of Rabat, Rabat, Morocco; 18 Vuk Vrhovac University Clinic for DiabetesEndocrinology and Metabolic Diseases, Merkur University Hospital, Zagreb, Croatia; 19 School of Medicine, Catholic University of Croatia, Zagreb, Croatia; 20 School of Medicine, Josip Juraj Strossmayer University of Osijek, Osijek, Croatia; 21 Faculty of Medicine, National Diabetic Center, University of Dakar, Dakar, Senegal; 22 Department of Internal Medicine, University of Medicine, Tirana, Albania

**Keywords:** Adolescents, Type 2 Diabetes, Prediabetes, Obesity, Prevention, Anti-obesity Medications, Artificial Intelligence, Digital Health.

## Abstract

**Background:**

The global rise in adolescent obesity is driving an aggressive phenotype of type 2 diabetes (T2D), which is marked by rapid progression and premature mortality. Therefore, evidence-based, internationally applicable prevention guidelines are urgently needed.

**Methods:**

A multidisciplinary international panel (*n* = 21) developed consensus recommendations through a systematic literature review (utilizing Web of Science, Scopus, PubMed, Cochrane, and EMBASE for the years 2000–2025) and a structured meeting process (held from October 2024 to December 2025). Consensus required ≥ 80% agreement. A total of eighty statements were initially proposed, from which sixty achieved preliminary agreement in Phase 1. Following iterative refinement in Phases 2 and 3, fifty achieved final consensus, and thirty were eliminated, either for failing to achieve 80% agreement or by being designated for further research.

**Results:**

The panel established fifty evidence-based recommendations across nine domains, which include screening, lifestyle intervention, school-based initiatives, digital health, therapeutic options, and policy. Key recommendations are to implement standardized screening protocols in schools, regulate the advertising of unhealthy foods and beverages, promote healthy lifestyle trends through media, and integrate artificial intelligence (AI) to tailor prevention plans.

**Conclusion:**

This consensus provides a practical, multisectoral framework for the global prevention of adolescent obesity and T2D, designed for application across healthcare, governmental, and non-governmental settings.

**Supplementary Information:**

The online version contains supplementary material available at 10.1186/s13098-026-02142-y.

## Introduction

The global surge in adolescent obesity is a critical health crisis, fueling a clinically aggressive form of type 2 diabetes (T2D) in this age group (10–19 years). While its etiology is multifactorial (involving lifestyle, genetic, and environmental factors), its prevalence has dramatically increased across high-, middle-, and low-income countries.

Urgent action is needed; current estimates indicate that well over 400 million children and adolescents were living with overweight or obesity in 2022. This number is projected to nearly double by 2030 if immediate intervention is not taken [1–3].

Insulin resistance, driven by obesity, precipitates prediabetes and T2D, alongside features of metabolic syndrome, including dyslipidemia, hypertension (HTN), acanthosis nigricans, ovarian hyperandrogenism, and metabolic dysfunction-associated steatotic liver disease (MASLD) [4].

The incidence of adolescent T2D has risen two-to-threefold over the past 30 years, with puberty being a particularly high-risk period [5]. This rise is not uniform globally. The most dramatic increases have been observed in the United States, China, and India [6], while rates in Europe, though growing, have been comparatively lower [7].

Registries from Germany [8], Hong Kong, India [9], and the Middle East and North Africa (MENA) region [10] have also contributed to the current knowledge.

T2D in adolescents has a worse clinical course than that in adults, involving early, rapid progression of cardiometabolic and microvascular complications and increased premature all-cause mortality [11]. Furthermore, these patients exhibit greater insulin resistance and reduced responsiveness to standard insulin sensitizers, with higher therapeutic failure rates [12].

Unfortunately, current guidelines lack a multidisciplinary framework that integrates the complex pathophysiology and provides tailored interventions for adolescents.

## Objectives

Collective efforts are needed to reduce the increasing rates of adolescent obesity and T2D. This consensus addresses adolescent obesity and T2D risk factors and aims to unify fragmented regional data, providing evidence-based recommendations for screening and novel tools for future prevention in this age group.

## Complications and comorbidities of adolescent obesity and t2d

Adolescents with overweight/obesity are more likely to remain obese in adulthood and experience noncommunicable diseases at a younger age. These individuals are at high risk for prediabetes, T2D, prehypertension, HTN, dyslipidemia, cardiovascular disease, and premature mortality in adulthood [13]. Other obesity-related comorbidities include obstructive sleep apnea, hyperandrogenism, and polycystic ovary syndrome (PCOS) [14].

The psychological complications of obesity include low self-esteem, depression, and discrimination by their community [15].

Adolescents who develop T2D experience a more rapid and progressive decline in beta-cell function [16] and are more likely to develop micro- and macrovascular complications, with early morbidity and mortality [17].

A meta-analysis of 26 observational studies comprising over a million individuals established an increased risk of macrovascular and microvascular disease and all-cause mortality associated with the onset of diabetes [18].

According to the TODAY study, at T2D diagnosis, 8% of patients had diabetic kidney disease (DKD), 19.2% had hypertension (HTN), 20.8% had dyslipidemia, and 13.7% had mild diabetic retinopathy. Fifteen years later, 80.1% of patients developed at least one microvascular complication, with rates rising dramatically: 54.8% DKD, 50% retinopathy, 35% peripheral neuropathy, and 67.5% HTN [19]. Cardiovascular disease is the major contributor to mortality even at young ages [20], at a rate much higher than that associated with type 1 diabetes [21].

## Methodology and consensus development

**(More methodology details are provided in the supplementary material.)**

- **Study Design**

This consensus project employed an Expert Panel Consensus Meeting Series following a systematic literature review to generate evidence-based, multisectoral recommendations for the global prevention and screening of obesity and T2D in adolescents aged 10–19 years.

- **Expert Panel**

The multidisciplinary international panel (*n* = 21) comprised experts in endocrinology, pediatrics, hepatology, nutrition, and internal medicine from the USA, Europe, Asia, and Africa. All members submitted conflict-of-interest disclosures.

- **Literature search**

A structured search of PubMed, the Cochrane Library, EMBASE, and Google Scholar (*January 2000–November 2025)* identified relevant studies.

- **Search Terms**: (“adolescent” OR “youth” OR “teenager”) AND (“obesity” OR “type 2 diabetes” OR “prediabetes”) AND (“prevention” OR “screening” OR “intervention”).
- **Inclusion criteria**: English-language publications; ages 10–19; and all study designs, including systematic reviews, randomized controlled trials (RCTs), observational studies, and clinical guidelines.
- **Exclusion**: Type 1 diabetes; adult-only populations.

**Evidence grading**

The evidence quality was assessed via GRADE (Grading of Recommendations Assessment, Development and Evaluation) methodology [22] (Table 1), which focuses on areas of low-quality evidence and the strength of recommendations.

**Table 1:** GRADE evidence quality criteria

Quality	Confidence	Study Design
High Quality	Very confident; true effect close to estimate	Well-conducted RCTs
Moderate Quality	Moderately confident; further research may impact estimate	RCTs with limitations or upgraded observational studies
Low Quality	Limited confidence: true effect may differ substantially	Well-conducted observational studies

RCTs: randomized controlled trials

- **Consensus process**

The consensus was established over two physical and thirty-eight virtual meetings (October 2024–December 2025) across three phases:

- **Phase 1** involved the distribution of eighty preliminary recommendations. Statements that did not achieve a consensus threshold of > 80% verbal agreement were eliminated, resulting in sixty statements.
- **Phase 2** focused on the iterative refinement of the sixty remaining statements, resolving issues related to feasibility, cost, and applicability through focused discussions.

Following Phase 2, ten statements still failed to achieve ≥ 80% agreement and were eliminated.

- **Phase 3** concluded with a final review and resolution of ambiguities, confirming fifty core recommendations, including conditional guidance for resource-limited settings.

**Consensus threshold**

The final recommendations required ≥ 80% agreement (agree/strongly agree). Statements below this threshold were documented as requiring further research.

## Screening

### Screening for obesity/overweight in adolescents

Annual body mass index (BMI) screening (weight/height², kg/m²) is recommended in schools, clinics, or community settings. Weight status is classified using the Centers for Disease Control and Prevention (CDC) growth charts, which are based on sex- and age-specific percentiles, as shown in Table 2.

**Table 2:** Weight status categories via CDC growth charts

Underweight	< 5th percentile
**Healthy weight**	from 5th to < 85th.
**Overweight**	from 85th to 95th.
**Obesity**	≥ 95th
**Severe obesity**	BMI is 120% or higher than the 95th percentile or ≥ 35 kg/m².

The CDC 2000 growth charts are used for adolescents with a BMI ≤ the 95th age- and sex-specific percentile, while the CDC 2022 extended charts are applied when BMI is > the 97th percentile. Either chart may be used for those between the 95th and 97th percentiles. The CDC online calculator is available for percentile determination and BMI classification in adolescents [23, 24]. In older adolescents, adult BMI cutoffs apply, with obesity defined as a BMI ≥ 30 kg/m² when the percentile is < 95th. BMI has known limitations, including overestimating obesity in individuals with edema or increased muscle mass and underestimating adiposity in Asians, who have higher body fat at lower BMIs than Europeans. Therefore, South and Southeast Asian populations should use country-specific growth charts or, if unavailable, International Obesity Task Force (IOTF) charts incorporating Asian data [25, 26].

However, BMI remains widely adopted for obesity screening because of its simplicity, cost-effectiveness, reproducibility, and correlation with direct adiposity measures (e.g., skinfold thickness, dual-energy X-ray absorptiometry) in most populations. BMI retains utility in tracking the response to weight-reducing interventions [27–30].

**Waist Circumference (WC)** is measured to assess central adiposity. Values above the 90th percentile for age and sex indicate an increased risk for metabolic complications [31] (Table 3).

*Recommendations for screening for obesity/overweight in adolescents*.

**Table 3:** Recommendations for screening for obesity/overweight in adolescents

Recommendation	Strength of Recommendation	Quality of Evidence
Annual BMI screening of adolescents. Use CDC 2000 charts if BMI ≤ 95th percentile, and CDC 2022 Extended charts if BMI > 97th percentile.	Strong^**†**^	Low
In Asians, use country charts if present or IOTF charts if not.	Strong	Low

BMI: Body mass index; ^**†**^Evidence-Recommendation Linkage is provided in the supplementary material

### Screening for T2D and prediabetes in adolescents

Most clinical practice guidelines, such as the Canadian Practice Guidelines 2018 [32] and the American Diabetes Association (ADA) 2026 [33], recommend risk-based screening for T2D/prediabetes in adolescents (Tables 4 and 5). Glycated hemoglobin (HbA1c), fasting plasma glucose (FPG), random plasma glucose, or 2-hr plasma glucose during a 75 g oral glucose tolerance test (2-hr OGTT) are the most commonly used methods. The Diabetes Canada 2018 guidelines recommend combining HbA1c with FPG/random plasma glucose to screen adolescents with risk factors for T2D. A 2-hr OGTT may be considered in the initial screening of adolescents with severe obesity, three or more risk factors, or conflicting results [32].

**Table 4:** Screening recommendations for T2D in adolescents were adopted from the Canadian Clinical Practice Guidelines (2018) [32]

Screening at-risk adolescents for T2D every 2 years with HbA1C and FPG/random plasma glucose testing:
a. Adolescents with ≥ 2 of the following risk factors: - Obesity. - Belonging to an ethnic group at high risk for T2D (e.g., African, Hispanic, Asian, Indigenous, Arab, or South Asian descent). - A first-degree relative with T2D and/or in utero exposure to hyperglycemia. - Features of insulin resistance (e.g., acanthosis nigricans, HTN, dyslipidemia, MASLD [ALT > threefold the upper normal level or fatty liver on ultrasonography]).
b. PCOS.
c. IFG and/or IGT.
d. Atypical antipsychotic drugs.

IFG: impaired fasting glucose, IGT: impaired glucose tolerance, ALT: alanine transaminase, HbA1C: glycated hemoglobin, FPG: fasting plasma glucose, 2-hr OGTT: 2-hr plasma glucose during a 75 g oral glucose tolerance test, HTN: hypertension, MASLD: metabolic dysfunction-associated steatotic liver disease, PCOS: polycystic ovary syndrome

**Table 5:** Screening recommendations for prediabetes/T2D in adolescents adopted from the ADA (2026) [33]

Screening asymptomatic adolescents for prediabetes/T2D starting post puberty or ≥ 10 years (whichever comes first) in those with obesity or overweight plus ≥ 1 risk factor:
- Maternal T2D or gestational diabetes in the adolescent’s gestation
- Family history (1st /2nd degree relative)
- High-risk ethnic groups (African American, Asian American, Native American, Latino, Pacific Islander)
- Signs of insulin resistance or associated conditions (acanthosis nigricans, hypertension, dyslipidemia, polycystic ovary syndrome, small-for-gestational-age birthweight).

*If the results are normal, screening is repeated every 2–3 years and at earlier intervals if an individual experiences weight gain or a deterioration of T2D risk factors. T2D: type 2 diabetes

**Table 6:** Diagnostic criteria for prediabetes/T2D diagnosis adopted from the ADA (2026) [33]

FPG	Prediabetes	T2D
	100–125 mg/dL (5.6–6.9 mmol/L)	≥ 126 mg/dL (≥ 7.0 mmol/L)
**Random plasma glucose**		≥ 200 mg/dL (≥ 11.1 mmol/L) in the presence of hyperglycemic symptoms
**2 h post-oral glucose in the 2 h OGTT**	140–199 mg/dL (7.8–11.0 mmol/L)	≥ 200 mg/dL (≥ 11.1 mmol/L).
**HbA1C**	5.7–6.4%	≥ 6.5%

FPG: fasting plasma glucose; 2-hr OGTT: 2-hr plasma glucose during a 75 g oral glucose tolerance test; HbA1C: glycated hemoglobin; T2D: type 2 diabetes

FPG alone may overdiagnose prediabetes/diabetes in adolescents [34], and fasting compliance may be challenging in this age group. OGTTs have a relatively high detection rate in severely obese or high-risk adolescents [35]. HbA1C ≥ 6.0% (performed via laboratory-based, DCCT-aligned, National Glycohemoglobin Standardization Program-certified methodology) has 86% sensitivity and 85% specificity for T2D/prediabetes, comparable to FPG when compared with **a** 2-hr OGTT, and offers convenience, as it does not require fasting. In insulin-resistant adolescents, the sensitivity and specificity increase to 99% and 96%, respectively. However, HbA1c results may conflict with those of glucose-based tests (FPG/OGTT), so combining tests is advised [32]. HbA1C limitations include inaccuracies in patients with hemoglobinopathies, anemia, renal failure, hemolysis, or from certain ethnicities; in these cases, OGTT/FPG are preferred. While HbA1c is practical, reliance on it alone is discouraged owing to variability. Guidelines emphasize the use of HbA1c alongside FPG/OGTT, particularly in high-risk youth, to balance accuracy and feasibility [33, 34, 36] (Tables 6 and 7).

*Recommendations for screening for T2D and prediabetes in adolescents*.

**Table 7:** Recommendations for screening for T2D and prediabetes in adolescents

Recommendation	Strength of Recommendation	Quality of Evidence
Screen adolescents with overweight or obesity and at least one additional risk factor for T2D or prediabetes.	Strong	Moderate
Combine HbA1C together with FPG or random plasma glucose as the initial screening tests.	Strong	Moderate
Consider a 2-hour OGTT for adolescents who have severe obesity, more than three risk factors, or inconsistent test results.	Conditional	Moderate
Combine HbA1C with FPG or OGTT In high-risk youth, to improve diagnostic accuracy while maintaining practicality.	Strong	Moderate
Repeat screening every 2–3 years if results are normal, and sooner if body weight increases or new risk factors emerge.	Strong	Moderate

FPG: Fasting plasma glucose, 2-hr OGTT: 2-hr plasma glucose during a 75 g oral glucose tolerance test, HbA1C: Glycated hemoglobin, T2D: Type 2 diabetes

## Dietary strategies

Every adolescent deserves a healthy start in life. Alterations in sleep timing and duration can influence nutritional and metabolic balance in a bidirectional manner [37–39]. Short-duration night sleep of less than 6 h changes hunger and appetite sensations, leading to increased body mass. Conversely, meal consumption in the evening is adversely linked to several sleep pattern variables. Additionally, consumption of a high glycemic index and fat-rich meals 4 h before sleep is associated with decreased sleep latency and higher values for body fat percentage [40–42].

The International Diabetes Federation (IDF) and World Health Organization (WHO) 2024 recommendations are to minimize screen time and sugar-sweetened beverage intake; encourage healthy eating patterns; adopt a healthy lifestyle **(**including adequate sleep duration and quality, avoiding tobacco and alcohol, and practicing emotional self-regulation); limit energy intake from total fats and sugars; increase consumption of fruits and vegetables, as well as legumes, whole grains, and nuts; consume unsweetened yogurt as a snack; and choose unsaturated fats [43–44].

Evidence confirms the inverse association between adhering to the Mediterranean diet (MedDiet) and obesity [45]. A recent meta-analysis assessed the associations among adherence to the MedDiet, BMI, and waist circumference (WC), revealing a protective effect against obesity in adolescents [46].

In teenagers, the daily caloric requirement is calculated based on age, sex, and activity status; in general, individuals aged 9 and older need 35 to 45 kcal/kg/day. Proteins from sources such as red and white meat, eggs, and legumes should be included. Essential fatty acids, including omega-3 and omega-6, are required for eicosanoid synthesis (prostaglandins and leukotrienes) and should be consumed in the diet; saturated fats should not exceed 10% of total intake [47]. Fiber improves digestive health and feeds healthy gut microbiota. Fruits and vegetables are the chief sources of fiber, and the recommendation is daily consumption of at least five servings; for adolescents, the goal fiber intake is 25 g for girls and 31 g for boys [48, 49].

The MedDiet is characterized by high consumption of plant-based foods such as fruits, vegetables, whole grains, legumes, seeds, and nuts, and olive oil; a moderate intake of fish; a low-to-moderate intake of dairy products; and a low intake of sweets and meat (especially red meat) [50]. The monounsaturated fatty acid (MUFA) diet prevents central body fat accumulation, decreases postprandial adiponectin expression, and decreases insulin resistance [51, 52]. Nuts are rich in unsaturated fatty acids, dietary fiber, antioxidants, polyphenols, proteins, vitamin E, and some trace elements, such as zinc and selenium [53].

The daily intake of at least five portions of fruits and vegetables rich in fiber reduces and slows the absorption of carbohydrates and lipids. Moreover, fiber has prebiotic effects, modifying the gut microbial composition and positively impacting body weight and metabolism [54].

Finally, fast eating is linked to increased body weight; in contrast, eating slowly promotes a better sense of fullness, more ghrelin suppression, and a stronger and more precise memory of the meal. Slow eating is associated with a 25% reduction in subsequent meal intake, which appears to be an effective approach for reducing food consumption [55] (Table 8).

*Dietary recommendations*.

**Table 8:** Dietary recommendations

Recommendation	Strength of Recommendation	Quality of Evidence
Set a good example for a healthy lifestyle. Adolescents are more likely to follow their parents’ example if they eat healthy foods and exercise often.	Conditional	Low
Family meals help promote healthy eating practices.	Conditional	Low
The last meal should be eaten at least 4 h before sleep, with 7–8 h of sleep per night.	Conditional	Low
Reduce screen time to less than 1–2 h daily and schedule it for the early afternoon.	Conditional	Low
Encourage adolescents to eat only when hungry, taking gradually over 20–30 min.	Conditional	Moderate
Avoid rewarding or punishing behaviors using food.	Strong	Low
Store fresh fruits, vegetables, and low-fat/fat-free milk in the refrigerator rather than unhealthy foods.	Strong	Moderate
Consume a minimum of five servings of fruits and vegetables daily.	Strong	Moderate
Include 2–3 dairy products each day.	Conditional	Low
Consume one egg daily.	Conditional	Low
Include fish, white meat, or at least two plant-origin proteins (beans, chickpeas, peanuts, lentils) four times/week.	Conditional	Moderate
Limit red meat to two times per week or replace it with at least two plant-origin proteins.	Conditional	Moderate
Consume nuts such as pumpkin seeds, watermelon seeds, peanuts, almonds, pistachios, and hazelnuts as snacks in moderate amounts (handfuls).	Strong	Moderate
Use Extra Virgin or Virgin Olive Oil as the main fat source, at approximately 10 ml/day.	Strong	Moderate
Avoid sugary beverages and choose water instead	Strong	High
Total sugar (white/brown) intake must never exceed 25 g/day; alternatively, use 1–2 teaspoons of pure honey; sugar substitutes are allowed in moderate amounts.	Strong	Moderate
Restrict processed and ultra-processed food.	Strong	Low

## Physical exercise

Physical activity is widely recognized to improve overall energy expenditure [56], and programs designed for weight management are advocated to include exercise training [57]. It improves body composition, BMI [58], metabolic syndrome components and insulin resistance in adolescents with obesity [59]. Similarly, insulin sensitivity is improved in individuals with prediabetes/T2D [60], as well as cardiorespiratory fitness [61] and psychosocial parameters [62].

The recommended exercise strategies involve recreational and systematized activities. Recreational activities include games, dancing, and swimming [63]. Systematized activities include running, resistance exercises, aquatic exercises, aerobic training, cycle ergometers, jump rope exercises, and a mix of sports (e.g., team sports, skiing and cycling) [64].

Exercise recommendations for adolescents include FITT exercise programs (frequency, intensity, time, and type), starting with 10 min of walking 3–5 days/week and increasing gradually to 60–80 min daily with an intensity of 55–90% of the maximum heart rate, using both aerobic and resistance modalities [65]. Many new technology devices and mobile applications, e.g., smartwatches, are useful for calculating heart rate, exercise intensity, and caloric expenditure.

Strength-based exercises, such as pushing and weightlifting, should be restricted during early adolescence and can be recommended at the end of somatic growth (late adolescence), while jumping or sprinting should be recommended instead [66].

Health organizations, including the ADA, IDF, the European Association of Cardiovascular Prevention and Rehabilitation, and the WHO, have recommended numerous and nearly similar physical activity programs for preventing obesity and T2D in adolescents.

The WHO recommends the following guidelines for physical activity to prevent obesity in adolescents: [67] adolescents should engage in 60 min of moderate- to vigorous-intensity daily physical activity, including aerobic and muscle- and bone-strengthening activities. Aerobic activities should be performed daily, whereas resistance exercises should be performed at least 3 times weekly. Activities should be enjoyable and varied to encourage sustained participation.

These exercise programs can be implemented in schools, universities, sports clubs, public youth sports, and fitness centers (Table 9).

*Recommendations for physical exercise*.

**Table 9:** Recommendations for physical exercise

Recommendation	Strength of Recommendation	Quality of Evidence
Exercise programs should be prescribed as one of the tools to prevent obesity and T2D for all, with a particular focus on adolescents with overweight and obesity.	Strong	High
Exercise programs can include recreational/systematized activities, with a recommendation of at least 60 min daily of moderate-to-vigorous intensity exercise.	Strong	High
Suggested activities include running, swimming, cycling, playing sports (such as football, basketball, etc.), jumping, climbing, and resistance exercises.	Strong	High
Strength exercises should be delayed until late adolescence.	Conditional	Low
Establishment of designed walking and cycling tracks and sports facilities in public areas to ensure widespread access to physical activity.	Conditional	Low

T2D: Type 2 diabetes

## School and university campaigns

To combat diabetes and obesity, schools and universities should promote healthy nutrition and physical activity. Mandating healthy cafeteria options, integrating nutrition education, and implementing initiatives such as “Fruit and Veggie Days” can foster healthier diets. WHO guidelines advise reducing the consumption of sugary drinks and increasing fruit/vegetable access [68]. Schools should enhance physical education, incorporate active breaks, and initiate programs to encourage walking or cycling to school.

IDF emphasizes integrating mental well-being into diabetes prevention, advocating institutional support to mitigate chronic stress, and promoting healthier student coping mechanisms [69]. Schools must address mental health through counsellors and stress management programs (e.g., mindfulness, yoga) to support students and create inclusive environments to reduce bullying.

Raising awareness among stakeholders is crucial. Training teachers and staff to recognize early signs of diabetes, conducting parent workshops on healthy habits, and collaborating with healthcare providers for education can increase prevention. The ADA emphasizes the need for community- and school-based diabetes awareness programs to foster a collaborative approach [70]. Moreover, the International Society for Pediatric and Adolescent Diabetes (ISPAD) has suggested improving diabetes and obesity literacy by implementing tailored education programs for students and staff [71].

School health programs, with parental consent, can screen for BMI, WC, and blood glucose levels to identify at-risk adolescents, enabling early referrals for professional medical care. National Organization for Health (NOH) guidelines suggest routine checks to track obesity trends and diabetes risk, supporting timely interventions [72].

Overcoming socioeconomic barriers is essential to ensure inclusivity in preventive measures. Collaborations with government agencies and nongovernmental organizations (NGOs) can help provide subsidized healthy meal programs and free or low-cost extracurricular physical activity programs. Awareness campaigns should address cultural misconceptions about body image and diabetes, while sharing success stories of students who adopt healthier lifestyles can inspire others to make positive changes.

The National Institute of Health (NIH) has emphasized integrating evidence-based programs such as “We Can!” (Ways to enhance children’s activity and nutrition) into school curricula, these programs focus on improving physical activity, nutrition, and screen-time behaviors in adolescents to lower T2D risk [73] (Table 10).

*Recommendations* *for school and university campaigns*.

**Table 10:** Recommendations for school and university campaigns

Recommendation			Strength of Recommendation	Quality of Evidence
Dealing with dietary habits, physical inactivity, stress, and socioeconomic and cultural challenges in schools and universities requires a comprehensive, multidisciplinary approach. Implementing targeted interventions can reduce the incidence of T2D and foster lifelong healthy habits among students.			Strong	Moderate
Integrate T2D and obesity prevention education into academic curricula.			Strong	Moderate
Planned exercise special programs tailored for students with overweight or obesity, and high-risk students should be held at schools			Strong	Moderate
**Protocol for Screening in Schools**	**Anthropometric measures** [68, 73,74] (weight, height, BMI, and WC).		Strong^**†**^	Low
	**Blood Pressure Monitoring**: Screen for high blood pressure, defined as systolic or diastolic readings ≥ 90th percentile for age, sex, and height [75].		Strong^**†**^	Low
	**Laboratory Testing** (for high-risk adolescents):	FPG, or random plasma glucose, and HbA1C [33]	Strong^**†**^	Low
		Lipid Profile (for high-risk adolescents) [76]	Strong^**†**^	Low
**Referral Criteria**: Refer students with obesity, abnormal WC, or any abnormal laboratory result to an endocrinologist or primary care physician for further evaluation.			Strong	Moderate
**Parental Involvement**: Provide parents with detailed results and educational materials, emphasizing the importance of lifestyle modifications.			Strong	Moderate

† Strong recommendation despite low-quality evidence is justified by (1) successful implementation in national screening programs (Japan, Singapore), (2) minimal risk of harm, (3) high potential for early detection in at-risk populations, (4) alignment with ADA, IDF, and ISPAD screening guidelines, and (5) critical public health need given rising adolescent T2D prevalence. More evidence-recommendation-linkage is provided in the supplementary material, Table S1

T2D: Type 2 diabetes, BMI: Body mass index, WC: Waist circumference, FPG: Fasting plasma glucose, HbA1C: Glycated hemoglobin

## Community campaigns

It is crucial to implement effective community-based interventions to prevent obesity and T2D in this vulnerable population [77]. The main determinants of the success of a community campaign are accessibility, affordability (if not totally free), and convenience for all citizens [78]. Community campaign strategies can play a significant role in preventing obesity and T2D among adolescents by promoting healthy lifestyle behaviors. Schools and university-based programs should integrate diabetes and obesity prevention education into curricula and foster a culture of healthy living within the academic environment.

To raise awareness, physical and virtual community workshops, seminars, and face-to-face interviews should be organized for parents, teachers, and coaches at locations such as schools, universities, sports clubs, malls, cafes, festivals, and concerts. Community initiatives to encourage the planting of fruit trees can help provide access to fresh fruits at low or no cost while promoting healthy eating habits. Likewise, community strategies to encourage physical activity, such as organizing marathons for all age groups, are essential for promoting healthy lifestyle behaviors (Table 11).

*Recommendations for community campaigns*.

**Table 11:** Recommendations for community campaigns

Recommendation	Strength of Recommendation	Quality of Evidence
Organize interactive events for all community sectors.	Conditional	Low
Encourage planting fruit trees outdoors and vegetables indoors.	Conditional	Low

## Media awareness

### Social media

Internet-based communication platforms that facilitate interactive content creation and sharing are increasingly essential. The most popular of these are Facebook, YouTube, WhatsApp, Instagram, TikTok, and X, which utilize features such as reels and hashtags [79]. The use of these platforms as sources of information and education has increased in popularity over the past two decades. More than 4.5 billion people use social media, including adolescents, who spend an average of 151 min daily on these platforms [80, 81]. Social media-based interventions supervised by healthcare authorities or scientific national diabetes and obesity societies can improve health behaviors [82].

Targeted social media campaigns to disseminate information about healthy lifestyles and weight management are particularly appealing for young age groups and, unexpectedly, among older age groups as well. Social media allows entire families to engage in online health initiatives.

These platforms provide easy access to health information, including videos, stories, cooking classes on balanced diets, portion control, mindful eating, and tips for exercise and stress management. Adolescents can find inspiration, motivation, and role models in Nobel Prize winners, influencers, celebrities, online forums, and supportive peer groups, alongside real-life examples and success stories of peers who have successfully managed their weight (Table 12).

## Conventional media

Messages delivered via radio or TV channels by national healthcare authorities reach a broad audience of caregivers and parents [83].

*Recommendations for media awareness*.

**Table 12:** Recommendations for media awareness

Recommendation	Strength of Recommendation	Quality of Evidence
Inspirational social and conventional media trends encourage people to adopt a healthy lifestyle for the prevention of obesity and T2D.	Conditional	Low

T2D: type 2 diabetes

## Role of artificial intelligence and advanced technology

Mobile applications with an educational component and feedback activity can be effective in reducing the attrition rate during lifestyle interventions to prevent adolescent obesity.

Mobile health technologies represent promising tools to support lifestyle counseling intensity in clinical practice. Moreover, these tools allow healthcare professionals to perform a multilevel approach that targets both parents and adolescents [84].

Artificial intelligence (AI)-based programs and applications are emerging as powerful tools in the prevention of obesity and T2D among adolescents; they are dynamic and in constant evolution, although ethical, legal, and technological deployment should be wisely managed in this vulnerable age group.

AI represents an innovative chronic disease care model, and collaboration between digital solutions and healthcare professionals and incentivizes the adoption of digital tools in data collection.

It provides customizable educational applications that address diverse users’ needs, combining general education with personalized guidance through videos, narration guides, and interactive lessons or games on how to measure weight, height, BMI, and WC, in addition to data entry of laboratory results, mainly low- and high-density lipoprotein (LDL, HDL), total cholesterol, triglycerides, FPG, and HbA1C [85].

AI customizes advice based on individual data logging, such as anthropometric measurements, meal logs, activities, and laboratory data. It provides data-driven insights and advanced risk prediction by analyzing user data, such as genetic markers, electronic health records, anthropometric measurements, and lifestyle trends such as diet and physical activity from wearable devices, to recommend actionable steps and motivate adherence through engagement tools such as AI-based virtual coaching and conversational agents (chatbots), which provide real-time feedback on physical activity, food intake, and weight loss [86, 87]. It sets goals, sends reminders and notifications for meals and stress management, and tracks progress to ensure alignment with health goals.

In cases of clinical or laboratory result abnormalities, AI-based applications can recommend contacting a healthcare professional for specific one-to-one interactive tailored advice or referring to a dietitian, psychologist, or trainer for personalized workout tips. Some applications can allow users to add comments or report mood status to help adolescents manage their stress and provide them with tips that raise their spirits. Dynamic video games using wireless handheld controllers can increase adolescents’ body movements and calorie expenditures.

Online platforms that use game design elements to encourage physical activity and healthy habits can be very appealing to this particular age group.

Research indicates that AI-powered applications, by engaging adolescents through technology they are familiar with, can increase their capacity for self-tracking and may lead to improved adherence to treatment protocols and better metabolic outcomes [86].

The integration of AI into adolescent health management offers significant potential for enhancing the overall public health infrastructure for prevention. It facilitates the analysis of large-scale, population-level health data, allowing for more strategic resource allocation and targeted community wellness. The privacy of users’ data should be guaranteed (Table 13).

*Role of artificial intelligence and advanced technology recommendations*.

**Table 13:** Role of artificial intelligence and advanced technology recommendations

Recommendation	Strength of Recommendation	Quality of Evidence
Digital solutions fed by data logs or AI can tailor prevention plans based on each user’s clinical, personal, and laboratory data.	Conditional	Low
They help monitor adherence to scheduled intervals and the achievement of goals set.	Conditional	Low
Digital applications will guide the adolescent to contact a healthcare professional in cases of abnormal results, or a health coach when needed.	Conditional	Low

Al: Artificial intelligence; T2D: Type 2 diabetes. Justification for Conditional Recommendations:

While AI-based interventions show promise in engaging adolescents through familiar technology platforms and demonstrate early evidence of effectiveness in digital health studies, the long-term RCT data specifically for adolescent obesity/T2D prevention remains limited. Conditional recommendations reflect the emerging nature of this field, potential concerns regarding data privacy and algorithmic bias, and the need for human oversight. These tools should complement, not replace, healthcare professional guidance

## Approved therapeutic options for obesity in adolescents

**Anti-obesity medications** provide a potential solution; however, their high cost may impede the use of some formulations.

**Orlistat** is approved by the Food and Drug Administration (FDA) and the European Medicines Agency for long-term obesity management in adolescents aged ≥ 12 years. It inhibits pancreatic and gastric lipase, reducing lipid absorption. Side effects include oily stools, fecal urgency/incontinence, and deficiency of fat-soluble vitamins, which may limit its use. Contraindications include chronic malabsorption and cholestasis [88]. According to the XENDOS study [89], orlistat reduces diabetes incidence by 37.3%, possibly by reducing weight and postprandial lipidemia [90].

**Phentermine** is FDA-approved for adolescents aged > 16 years for a short duration of up to 12 weeks. It has potential anti-obesity effects, including reducing the reuptake of norepinephrine [91–93]. The side effects include irritability, insomnia, mood alteration, dry mouth, dizziness, tremors, and elevations in heart rate and blood pressure. No serious adverse effects were noted in studies on adolescents. Contraindications include a history of cardiovascular disease, hyperthyroidism, glaucoma, and the current use of monoamine oxidase inhibitors [92].

**Glucagon-like peptide-1 (GLP-1) receptor agonists (liraglutide and semaglutide)** are chemically modified mimetics of the incretin hormone GLP-1 that reduce weight via their central, hypothalamic action to suppress appetite and promote satiety. They also inhibit postprandial gastric emptying, thereby reducing appetite and inducing satiety [94]. Liraglutide and semaglutide are FDA-approved for treating obesity in adolescents aged 12 years and older. The side effects include nausea, vomiting, and gastroenteritis. They are contraindicated in patients with a family history of medullary thyroid cancer or multiple endocrine neoplasia type 2.

The once-daily recommended dose of liraglutide is 0.6 mg, increased gradually to 3.0 mg weekly to avoid gastrointestinal side effects [93–95]. The SCALE study revealed that only 3% of prediabetic individuals with overweight/obesity treated with liraglutide developed diabetes compared to 11% in the placebo group [96].

**Semaglutide**, a long-acting agent, allows once-weekly 2.4 mg dosing [97]. It significantly reduced weight and cardiometabolic risk factors in the 2022 STEP Teens study [98], significantly reduced total cholesterol, LDL, triglyceride, and ALT levels, and significantly lowered the 10-year T2D risk in individuals with overweight or obesity in STEPs 1 and 5 [99].

**Tirzepatide**, is a dual GLP-1 and a glucose-dependent insulinotropic polypeptide (GIP) receptor agonist, is the most effective available agent for glycemic control and weight loss [100], resulting in an average weight loss of 26% [101]. It reduces the risk of developing T2D by 94% in people with prediabetes and overweight/obesity [88]. It is FDA-approved for patients starting from 18 years of age.

### Phentermine-topiramate combination

This combination, FDA approved in 2022 for weight management in patients aged 12 years and older, addresses specific limitations of the two drugs when used individually. Phentermine is a sympathomimetic that can cause side effects such as tachycardia, insomnia, and anxiety, along with concerns regarding long-term efficacy and misuse potential. Topiramate monotherapy required high doses to induce significant weight loss, which could lead to severe adverse events, including cognitive impairment, paresthesia, and metabolic acidosis. A low-dose combination therapy provides synergistic and safer weight reduction [102, 103] and reduces the incidence of new-onset T2D over two years [104].

### Bariatric surgery

This should be considered cautiously after the failure of alternative therapies in adolescents with severe obesity who are 13 years or older or in adolescents with T2D, after weighing the advantages and risks. Preoperative and postoperative care should be provided by a multidisciplinary team [105] (Table 14).

*Recommendations for pharmacotherapy and bariatric surgery in adolescents with obesity or overweight*.

**Table 14:** Recommendations for pharmacotherapy and bariatric surgery in adolescents with obesity or overweight

Recommendation	Strength of Recommendation	Quality of Evidence
Orlistat, liraglutide, and semaglutide are used for managing obesity in adolescents starting at age of 12.	Strong	High
Phentermine can be used starting from 16 years old	Conditional	Low
Tirzepatide can be used starting at age of 18.	Strong	High
Adolescents aged 13 and older with severe obesity are referred for evaluation for metabolic and bariatric surgery at regional, comprehensive, multidisciplinary bariatric surgical centers.	Strong	Low

Local, national, or regional recommendations are applied for managing adolescents with obesity via anti-obesity medications or bariatric surgery after referral to specialized consultants or centers.

## Initiatives for policy makers

The role of governments in endorsing prevention policies is illustrated in Table 15.

**Table 15:** Recommendations for policymakers

Recommendation	Strength of Recommendation	Quality of Evidence
Governments should oblige food producers to label each product with detailed information regarding the following: a. Calorie content b. Nutritional contents c. Type and quantity of added sweeteners d. Type and quantity of fat content	Strong^**†**^	Low
Health care authorities should encourage consumers to read the information on food products.	Strong^**†**^	Low
Governments should impose high taxes on sweetened beverages and energy drinks.	Strong^**†**^	Low
Governments should declare power drinks as age-restricted drinks for those over 20.	Strong^**†**^	Low
Governments should control the advertisement of processed foods, sweetened beverages, and power drinks and promote advertising for healthy food.	Strong^**†**^	Low

^**†**^Strong recommendations for policy interventions are based on (1) substantial observational evidence from sugar-sweetened beverage taxation in Mexico, the UK, and other countries showing reduced consumption; (2) WHO recommendations on marketing restrictions; (3) successful analogous policies in tobacco control; and (4) the ethical imperative to protect vulnerable adolescent populations from predatory marketing, despite the absence of RCT-level evidence, which is not feasible for policy interventions. More evidence-recommendation-linkage is provided in the supplementary material, Table S1

## Ethical considerations

- **Consent**:

Before screening, comprehensive written parental/guardian consent must be obtained. This consent must include a clear explanation of the purpose, procedures, potential benefits, and risks, and it must state that participation can be declined without penalty. Screening results must be communicated confidentially to parents/guardians and adolescents with clear guidance on next steps.

- **Data Privacy and Governance**.

The integration of AI and digital health tools raises significant data privacy concerns, particularly for minors. All digital platforms must comply with applicable data protection regulations (e.g., Children’s Online Privacy Protection Act (COPPA)) and ensure:

- Informed consent for data collection.
- Data encryption with access limited to authorized healthcare providers.
- Right to data deletion upon request.

- Risk of Stigmatization and Psychosocial Harm

Obesity and diabetes screening carry inherent risks of stigmatization, bullying, and psychological distress, particularly in school settings. To mitigate these harms:

- Screening should be conducted in private, confidential settings.
- Results must never be disclosed publicly.
- Educational campaigns must emphasize health rather than appearance, avoiding weight-shaming language.
- Mental health support must be integrated into all screening and intervention programs.
- Interventions should be framed positively (e.g., “healthy lifestyle programs”) rather than punitively.

- Equity and Access

Prevention strategies must not exacerbate existing health inequities. Interventions should be designed to be accessible and affordable across socioeconomic groups, with subsidized or free programs for disadvantaged populations. Policy recommendations (e.g., taxation) must be evaluated for potential regressive impacts on low-income families, with compensatory measures (e.g., subsidies for healthy foods) implemented where appropriate.]

- **Autonomy and non-maleficence**.

While strong recommendations are made for prevention, all interventions must respect adolescent and family autonomy. Coercive approaches to weight management are ethically unacceptable and counterproductive. Healthcare providers must employ shared decision-making, exploring adolescent and family values, preferences, and barriers to healthy behaviors.

Pharmacotherapy and bariatric surgery recommendations must be approached with caution, ensuring thorough informed consent, a discussion of risks and benefits, and an exploration of less invasive alternatives before proceeding (Table 16).

**Table 16:** Ethical Principles for Implementation. Core ethical safeguards for adolescent obesity and T2D prevention programs

Recommendation	Strength of Recommendation	Quality of Evidence
Obtain explicit parental consent and adolescent assent (age ≥ 12) before school-based screening.	Strong^**†**^	Low
Ensure all digital health platforms comply with data protection regulations and have transparent privacy policies.	Strong^**†**^	Low
Conduct screening in private, confidential settings to minimize stigmatization.	Strong^**†**^	Low
Integrate mental health support into all prevention programs.	Strong	Moderate
Design interventions to be accessible across socioeconomic groups with subsidized options for disadvantaged populations.	Strong^**†**^	Low
Employ shared decision-making in all therapeutic interventions, particularly for pharmacotherapy and surgery.	Strong	Moderate

Ethical Imperative: These strong recommendations reflect fundamental ethical principles in adolescent healthcare that must be upheld regardless of evidence quality. Grounded in:

Autonomy and respect for persons: Adolescents are a vulnerable population requiring special protections

Non-maleficence: Prevention programs must not cause psychological or social harm

Justice: Interventions must be equitable and accessible to all socioeconomic groups

Beneficence: Mental health integration and shared decision-making optimize outcomes

^**†**^Strong recommendations despite low-quality evidence are justified by ethical necessity, professional consensus, alignment with international pediatric ethics guidelines (AAP, WHO), and potential for significant harm if these protections are not implemented

## Discussion

This consensus addresses socioeconomic disparities, recognizing that targeted interventions must differ between low-income and affluent communities to achieve global feasibility. The implementation of preventive strategies is determined by national policies, regulations, income, and individual socioeconomic status.

We offer novel contributions and major strengths:

- The integration of specific school- and university-based screening protocols empowers nonspecialist settings to participate in early detection.
- Recommendations are adaptable to various cultural and genetic backgrounds.
- The incorporation of AI is proposed as a core component of future prevention strategies.
- Additionally, the consensus offers a comprehensive “multilevel prevention framework” that uniquely provides policy interventions alongside clinical care, bridging the gap between public health and clinical practice.

### Limitations

First, a formal cost-effectiveness analysis for the proposed interventions (especially novel pharmacotherapy and AI tools) was not conducted. Second, regional disparities in the availability of high-quality RCTs necessitate reliance on observational data. Third, significant investment is needed to train primary care providers and school nurses in the proposed screening algorithms.

A critical and overarching limitation is the need for careful cultural adaptation of several core recommendations.

Dietary Recommendations: Our endorsement of dietary patterns like the Mediterranean diet is based on strong evidence, but its specific components (e.g., olive oil, specific fish, or daily servings of fresh fruits and vegetables) may be inaccessible or unaffordable. Recommendations must be translated into culturally appropriate, locally available foods to ensure adherence and sustainability.

Physical Activity Guidelines: Norms around physical activity, especially for adolescent girls, vary significantly across cultures due to religious practices and gender norms.

Socioeconomic Intersection: These cultural factors are inextricably linked to socioeconomic status. In low-resource settings, the primary barrier may not be cultural preference but a systemic lack of access to healthy foods, safe recreational spaces, or healthcare infrastructure.

Our policy recommendations, while vital, may be challenging to implement where regulatory frameworks are weak or where the food environment is dominated by ultra-processed products.

This consensus recognizes heterogeneities across countries and the feasibility for application, especially in low- and middle-income countries; therefore, our recommendations are adaptable guiding principles, not prescriptive mandates, tailored to the local context. Successful implementation will require partnership with local stakeholders, including community leaders, educators, and healthcare workers, to adapt strategies in a way that respects cultural contexts while preserving core scientific integrity.

## Conclusion

This consensus provides a vital, actionable roadmap for combating the global epidemic of adolescent obesity and T2D. By embracing a multisectoral approach that spans clinical, educational, digital, and policy domains, it charts a course for effective prevention. The aggressive nature of adolescent T2D demands urgent and coordinated implementation of these strategies. To translate this framework into tangible health improvements, robust international collaboration among governments, healthcare systems, non-governmental organizations, and the private sector is imperative. Success depends on a shared commitment to creating healthier environments for youth everywhere.

## Supplementary Information

Supplementary Material 1

## Data Availability

No datasets were generated or analysed during the current study.

## Abbreviations

AASD: Arabic Association for the Study of Diabetes and Metabolism
ADA: American Diabetes Association
AI: Artificial intelligence
ALT: alanine transaminase
BMI: Body mass index
CDC: Centers for Disease Control and Prevention
COPPA: Children’s Online Privacy Protection Act
DKD: Diabetic kidney disease
FDA: Food and Drug Administration
FITT: Frequency, Intensity, Time, and Type
FPG: Fasting plasma glucose
GIP: Glucose-dependent insulinotropic polypeptide
GLP-1: Glucagon-like peptide-1
GRADE: Grading of Recommendations Assessment, Development and Evaluation
HbA1C: Glycated hemoglobin
HDL: high-density lipoprotein
HTN: Hypertension
IDF: International Diabetes Federation
IFG: Impaired fasting glucose
IGT: Impaired glucose tolerance
IOTF: International Obesity Task Force
ISPAD: International Society for Pediatric and Adolescent Diabetes
LDL: Low-density lipoprotein
MENA: Middle East and North Africa region
MASLD: metabolic dysfunction-associated steatotic liver disease
MedDiet: Mediterranean Diet
MUFA: Monounsaturated Fatty Acid
NGOs: Non-Governmental Organizations
NIH: National Institute of Health
NOH: National Organization for Health
OGTT: Oral glucose tolerance test
PCOS: polycystic ovary syndrome
RCTs: randomized controlled trials
T2D: Type 2 diabetes mellitus
US: United States
VM: virtual meeting
WC: Waist circumference
WHO: World Health Organization
