# Supplementary material for: International consensus on adolescent metabolic health: prevention of obesity and type 2 diabetes
Source: Diabetol Metab Syndr. 2026 Apr 18;18:103. doi: 10.1186/s13098-026-02142-y (PMC13112707; doi:10.1186/s13098-026-02142-y)

**Supplementary material**

**METHODOLOGY AND CONSENSUS DEVELOPMENT**

- **Expert Panel**
- Panel members were selected based on (1) recognized expertise in adolescent metabolic health, obesity, or diabetes (defined as ≥5 years of clinical/research experience and ≥10 peer-reviewed publications in relevant fields), (2) geographic diversity to ensure global applicability, (3) representation from high-, middle-, and low-income countries, and (4) multidisciplinary expertise spanning clinical care, public health, nutrition, and policy.
- **Geographic distribution:** MENA region (n=10), Europe (n=5), Asia (n=3), Africa (n=2), and North America (n=1). While MENA representation was intentionally higher, given the consensus lead organization (AASD), all regions had proportional representation, which is based on the global burden of adolescent T2D.
- **Handling of disagreements:** When statements did not achieve ≥80% agreement, the following process was employed: (1) open discussion of the specific concerns raised by dissenting members, (2) review of additional supporting or contradicting evidence, (3) revision of statement wording to address concerns while preserving intent, and (4) re-voting after revision. Statements that failed to achieve consensus after two revision cycles were either eliminated or documented as requiring further research.
- **Alternative methodologies considered**: A formal Delphi process was considered but not employed due to (1) the need for real-time adaptation to diverse regional contexts, (2) time constraints given the urgency of the adolescent obesity epidemic, and (3) the collaborative nature of the consensus requiring extensive discussion rather than isolated voting rounds. We acknowledge this methodological choice and have positioned this work as expert consensus guidance based on systematic evidence review rather than a formal clinical practice guideline.”
- **Literature search**

“Google Scholar was included to capture grey literature, conference proceedings, and international guidelines that may not be indexed in traditional databases, particularly from low- and middle-income countries. However, to maintain quality, only the first 1,200 results (sorted by relevance) were screened, and all included Google Scholar sources were verified for credibility (peer-reviewed journals, official health organization reports, or government documents).

**Inclusion criteria:** For intervention studies, both efficacy and effectiveness studies were included. For screening recommendations, validation studies and implementation research were prioritized.

**Exclusion:** editorials and commentaries without original data; studies published before 2000; non-English publications without validated translations; animal studies; and studies with insufficient methodological detail to assess quality.”

**FIGURE 1: PRISMA Flow Diagram for Literature Review**


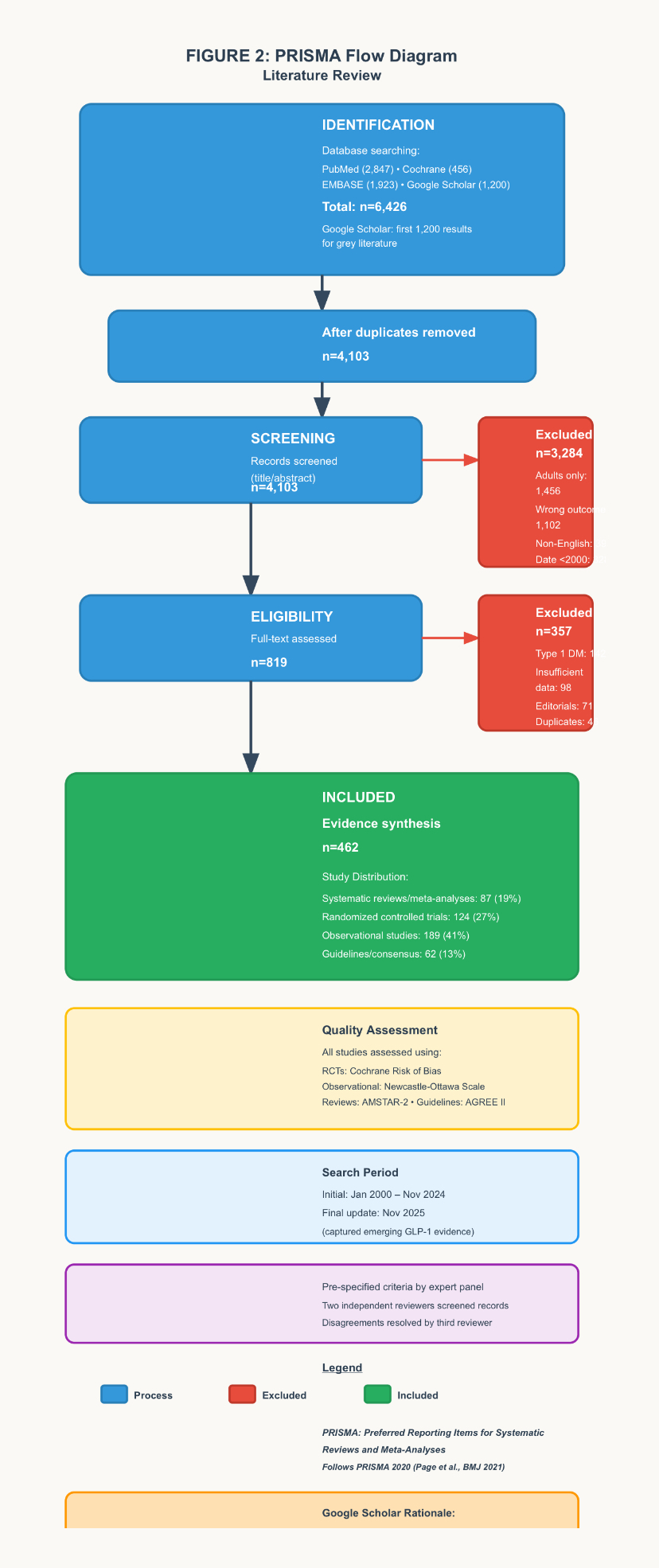


- **Evidence grading**

According to GRADE methodology, the strength of a recommendation reflects the extent to which we can be confident that the desirable effects of an intervention outweigh the undesirable effects. While high-quality evidence typically supports strong recommendations, GRADE explicitly allows strong recommendations based on low-quality evidence when: (1) the intervention addresses a critical public health problem, (2) there is strong indirect evidence of benefit, (3) the balance of benefits to harms is substantial, and (4) patient/population values strongly favour the intervention despite uncertainty.

- **Consensus process**

We acknowledge that openly and verbally conducted voting carries a potential for dominance bias. This methodology was chosen to enable immediate clarification of ambiguities and collaborative refinement based on diverse regional perspectives across the international panel. To mitigate conformity bias, all panel members were explicitly encouraged to voice dissenting opinions, and the chairpersons actively solicited input from all participants, particularly those from underrepresented regions. Senior and junior experts were given equal opportunity to contribute. Each statement underwent multiple rounds of discussion before voting, allowing sufficient time for critical evaluation. We recognize that anonymized Delphi methodology may have reduced potential bias; however, our approach prioritized the synthesis of diverse clinical experiences across six continents and facilitated the adaptation of recommendations to varied resource settings, which required extensive real-time dialogue.

- **Phase 1** involved the distribution of eighty preliminary recommendations. Statements that did not achieve >80% verbal agreement were either eliminated (n=20) or marked for revision (n=60), resulting in sixty statements advancing to Phase 2.
- **Phase 2** focused on the iterative refinement of the **[sixty]** remaining contentious statements, resolving issues related to feasibility, cost, and applicability through focused discussions. Following Phase 2, ten statements still failed to achieve ≥80% agreement and were eliminated.
- **Phase 3** concluded with a final review and resolution of ambiguities, confirming fifty core recommendations, including conditional guidance for resource-limited settings.
- **Consensus threshold**

The agreement threshold (≥80%) of the final recommendations was selected based on established consensus methodology standards used in similar international clinical practice guidelines (e.g., GRADE approach, Delphi methodology), which typically employ thresholds ranging from 70-80% for strong consensus. The 80% threshold was chosen to ensure robust expert agreement while maintaining practical feasibility for global implementation across diverse healthcare settings. This level balances the need for substantial consensus with the recognition that some variation in clinical practice and resource availability exists across the represented regions (MENA, Europe, Asia, Africa, and North America).

**
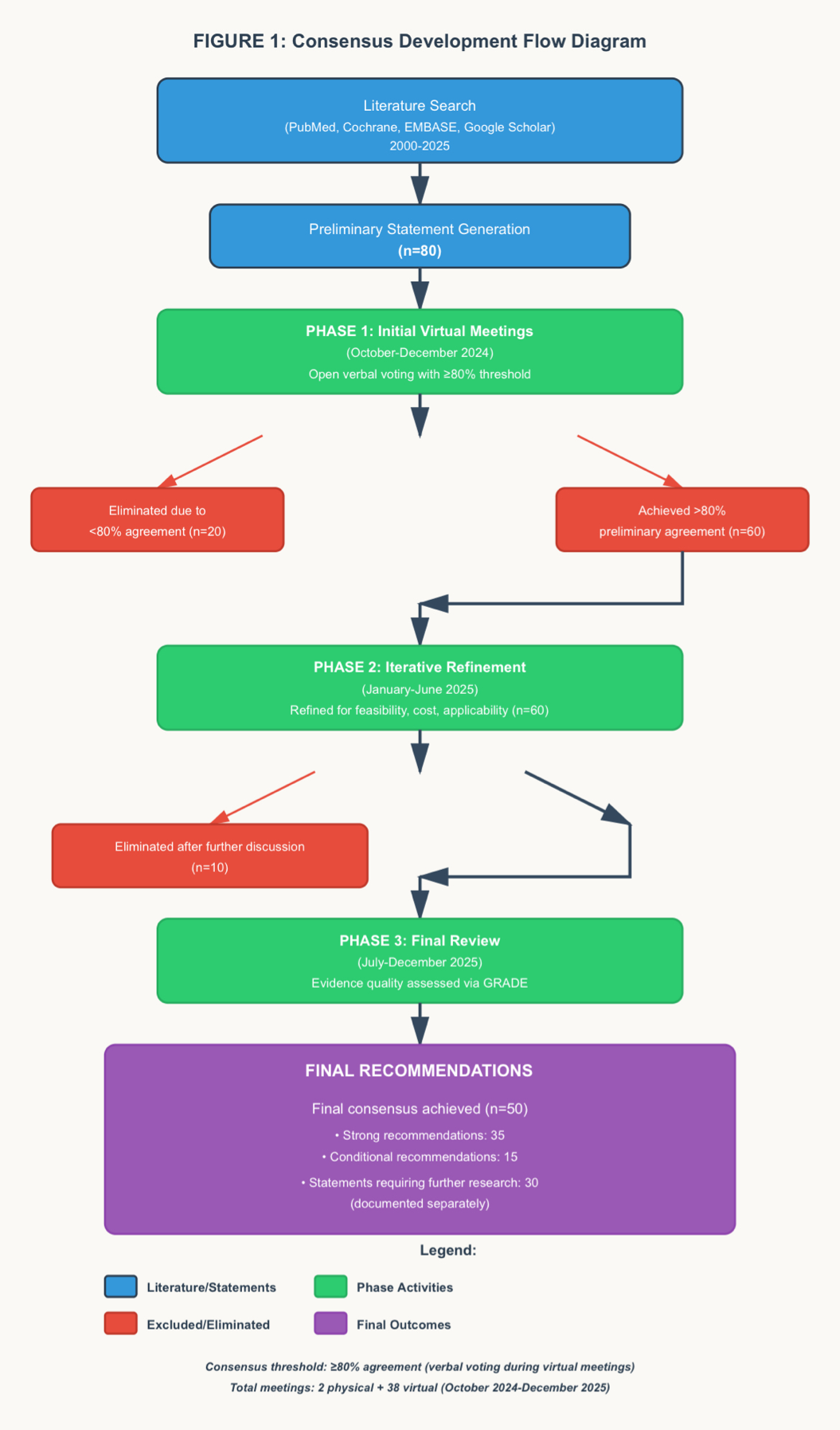
FIGURE 2: Consensus Development Flow Diagram**

**Supplementary Table S1. Evidence-Recommendation Linkage for Key Recommendations**

| **Screening Recommendations** |
| --- |
| 1. **Annual BMI screening of adolescents using CDC growth charts**   **Key Supporting Evidence**:   - CDC Clinical Growth Charts [23] - AAP Clinical Practice Guideline (Hampel et al., Pediatrics 2023) [24] - Population screening data from Japan Ministry of Health - Singapore Health Promotion Board school screening program - Javed et al. meta-analysis on BMI diagnostic performance (Pediatric Obesity 2015) [27]   Note: Evidence is primarily observational; no randomized controlled trials (RCTs) directly comparing screening vs. no screening are available.  **GRADE Quality:** Low Quality  (Based on observational data and expert consensus)  **Justification for Strong Recommendation** (despite low-quality evidence):   - Minimal risk and harm from BMI measurement - Low cost and high feasibility - High potential benefit from early identification of overweight/obesity - Successfully implemented in multiple national programs - Endorsed by major organizations: AAP, ADA, WHO, IDF - Critical public health need due to rising prevalence of type 2 diabetes (T2D) - Strong indirect evidence from cardiovascular disease (CVD) screening programs   **Alternative Perspectives Considered**:   - Concern about stigmatization (addressed via privacy protocols) - BMI limitations in populations with high muscle mass (acknowledged; use clinical judgment) - Resource constraints in low-income settings (conditional approach recommended in those contexts) |
| 1. **School-based laboratory screening (HbA1c, fasting plasma glucose – FPG) for high-risk adolescents**   **Key Supporting Evidence**:   - Canadian Diabetes Association Clinical Practice Guidelines 2018 [32] - ADA Standards of Care 2026 [33] - Buse et al. HbA1c validation study (Diabetes Care 2013) [36] - Implementation data from school programs in Japan, Singapore, UAE - TODAY Study screening protocols [19] - Note: RCT evidence unavailable; based on observational studies and expert consensus.   **GRADE Quality:** Low Quality  **Justification for Strong Recommendation** (despite low-quality evidence):   - successful implementation in national screening programs (Japan, Singapore), - minimal risk of harm, - high potential for early detection in at-risk populations, - alignment with ADA, IDF, and ISPAD screening guidelines, - critical public health need given rising adolescent T2D prevalence. |
| **Policy-Level Interventions** |
| 1. **Governments should impose high taxes on sweetened beverages and power drinks**  - **Key Supporting Evidence**: - Mexico SSB tax: Colchero et al., BMJ 2016 – 12% reduction in purchases after 2 years - UK sugar levy: Public Health England 2020 – reformulation of 50% of products - Berkeley, CA tax: Lee et al., AJPM 2019 – 52% reduction in SSB consumption - Cochrane Review: Pfinder et al. 2020 – price increases reduce consumption - WHO recommendations on fiscal policies (2022) - Note: Evidence comes from observational/ecological studies and natural experiments; RCTs are not feasible for policy-level interventions. - **GRADE Quality**: Low Quality (Observational data from natural experiments) - **Justification for Strong Recommendation** (despite low-quality evidence) - Substantial observational evidence of effectiveness - Successful analogous policies (tobacco, alcohol) - Endorsed by WHO and multiple national health agencies - Magnitude of the obesity epidemic requires population-level intervention - Ethical imperative to protect vulnerable adolescents from predatory marketing - Minimal direct harm; benefits likely outweigh costs - RCT-level evidence is inherently unavailable for policy interventions - **Alternative Perspectives Considered**: - Regressive impact on low-income families (addressed via food subsidies recommendation) - Industry opposition (acknowledged but public health prioritized) - Questions about long-term effectiveness (monitoring recommended) |
| 1. **Governments should regulate advertising of processed foods to adolescents**  - **Key supporting evidence**: - Who set of recommendations on the marketing of foods and non-alcoholic beverages to children (2010, updated 2023) |

**
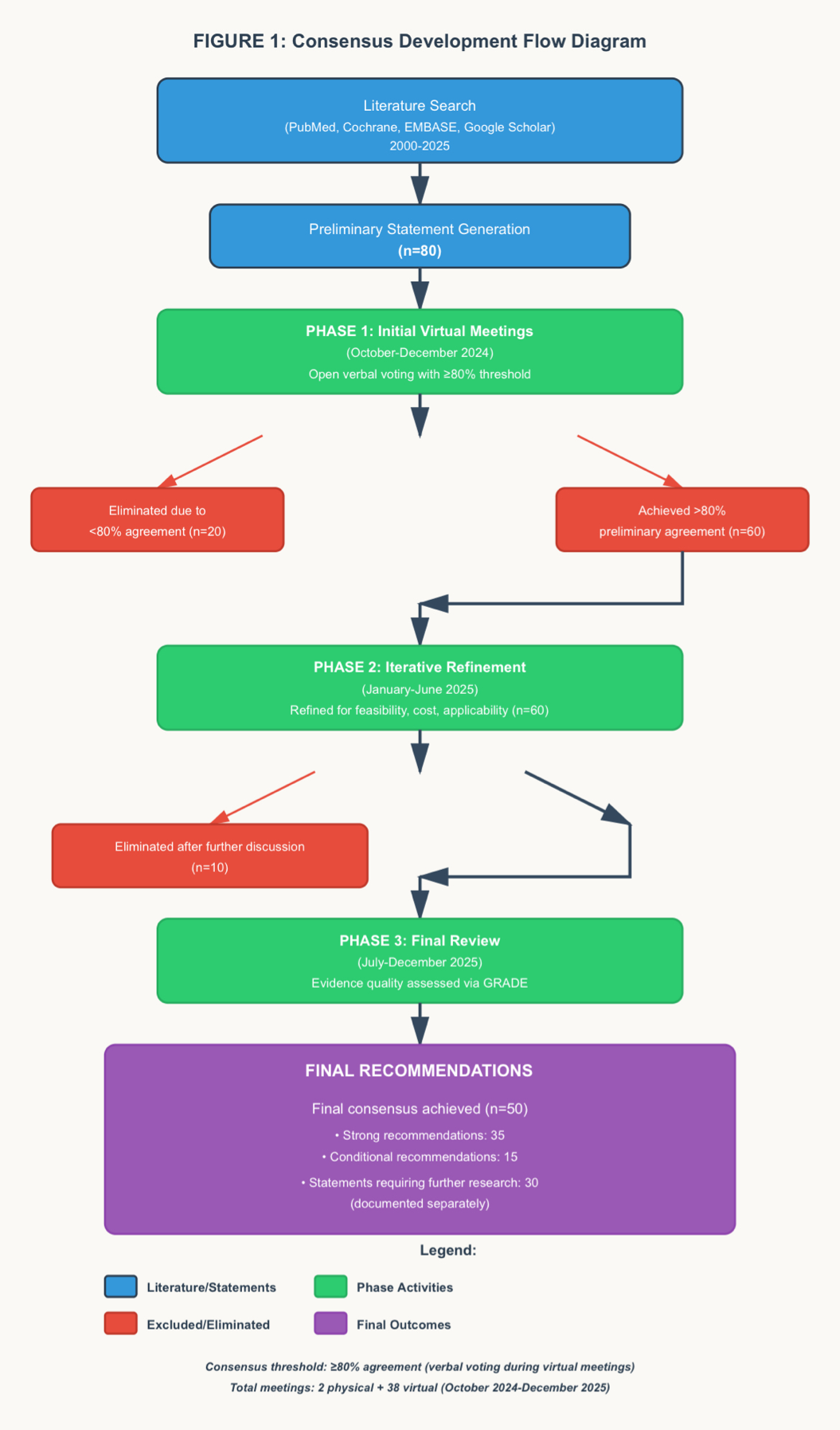
FIGURE 1: PRISMA Flow Diagram for Literature Review**


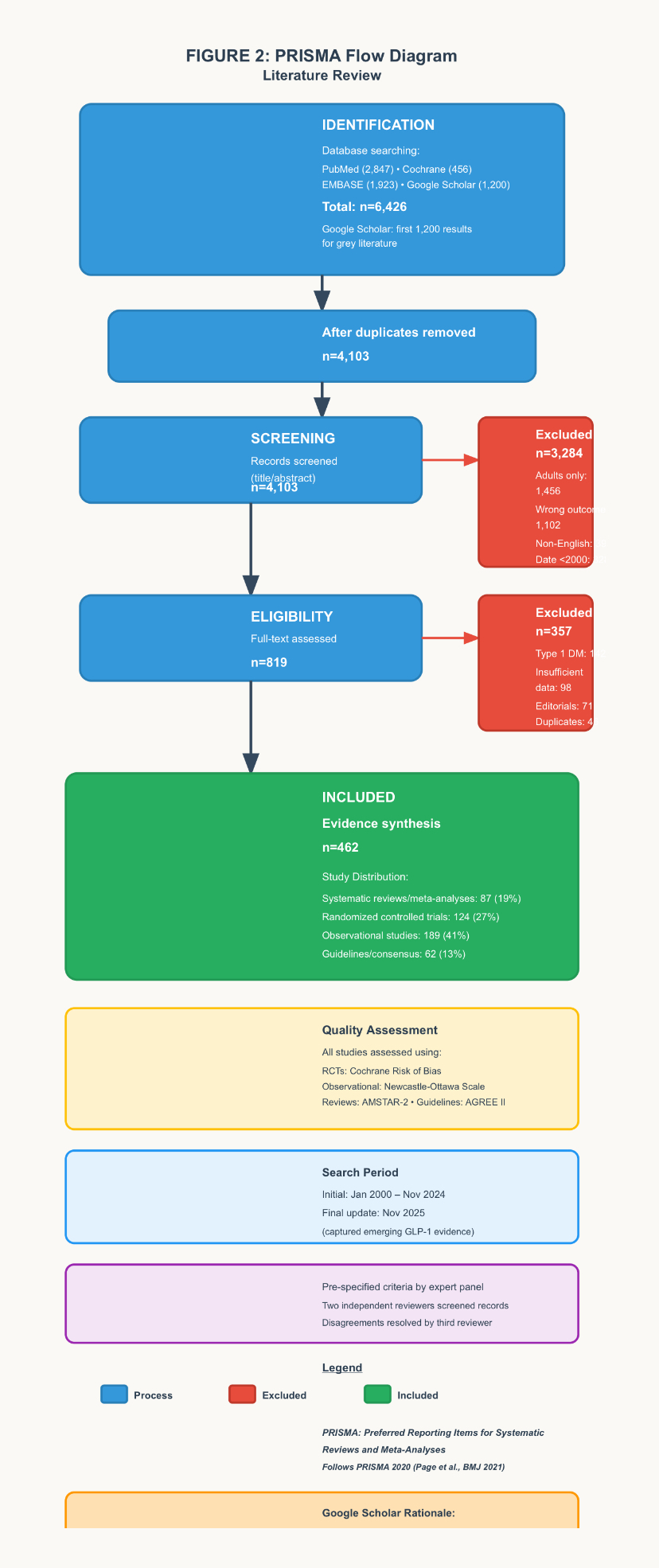

Supplement: Supplementary file 1 — Supplementary Material 1 [file 13098_2026_2142_MOESM1_ESM.docx]
